# The Role of IL-36 in the Pathophysiological Processes of Autoimmune Diseases

**DOI:** 10.3389/fphar.2021.727956

**Published:** 2021-10-05

**Authors:** Wen-jian Chen, Xiao Yu, Xin-Rong Yuan, Bang-jie Chen, Na Cai, Shuo Zeng, Yuan-song Sun, Hai-wen Li

**Affiliations:** ^1^ Department of Orthopaedics, Anhui Provincial Children’s Hospital, Hefei, China; ^2^ Inflammation and Immune Mediated Diseases Laboratory of Anhui Province, School of Pharmacy, Anhui Medical University, Hefei, China; ^3^ Department of Neurology, Xiangya Hospital, Central South University, Changsha, China; ^4^ The First Clinical Medical College of Anhui Medical University, Hefei, China; ^5^ Department of Emergency Surgery, The Second Hospital of Anhui Medical University, Hefei, China; ^6^ Department of Gastroenterology, The Third Affiliated Hospital of Anhui Medical University, Hefei, China; ^7^ Department of Gastroenterology, The First Affiliated Hospital of Zhengzhou University, Zhengzhou, China

**Keywords:** IL-36, autoimmune diseases, pro-inflammatory, interleukins, inflammatory responses

## Abstract

A member of the interleukin (IL)-1 superfamily was IL-36, which contained IL-36α, IL-36β, IL-36γ, and IL-36Ra. Heterotrimer complexes, consisting of heterodimeric receptor complexes and IL-36 agonist, gave signals through intracellular functional domains, so as to bind to downstream proteins and induce inflammatory response. IL-36 agonists upregulated mature-associated CD80, CD86, MHCII, and inductively produced several pro-inflammatory cytokines through the IL-36R-dependent manner in dendritic cells (DCs). Besides, DCs had the ability to initiate the differentiation of helper T (Th) cells. Up to date, the role of IL-36 in immunity, inflammation and other diseases is of great importance. Additionally, autoimmune diseases were characterized by excessive immune response, resulting in damage and dysfunction of specific or multiple organs and tissues. Most autoimmune diseases were related to inflammatory response. In this review, we will conclude the recent research advances of IL-36 in the occurrence and development of autoimmune diseases, which may provide new insight for the future research and the treatment of these diseases.

## Introduction

IL-36 was originally identified as a new member of the IL-1 superfamily in the process of DNA database screening for IL-1 homologs. Abreast researches confirmed that IL-36 cytokine was expressed in keratinocytes (KCs) and various immune cells including dendritic cells (DCs), T cells, and B cells. (Table 1) (Mai et al., 2018) In fact, in early pregnancy, IL-36 subtypes could be regulated differentially in the same tissue. For example, the expression level of IL-36γ was increased in luminal and glandular epithelium, while that of IL-36α and IL-36β were decreased (Murrieta-Coxca et al., 2019) Of note, there was a current evidence that IL-36 may act as a bridge between inflammation and fibrosis (Elias et al., 2021) The role of IL-36 evolved a variety of regulation, for example, it could provide effective and maximum anti-inflammatory effects by corresponding to IL-17 and IL-38 (Gabay and Towne, 2015).

**TABLE 1:** Overview of the IL-36 cytokines.

IL-36 cytokines	Previous names	Expression cells	References
IL-36α	IL-1F6	Monocytes, T/B cells	29,416,822
			29,571,080
			31,736,959
IL-36β	IL-1F8	Monocytes, T/B cells	29,416,822
			29,571,080
			31,736,959
IL-36γ	IL-1F9	KCs, epithelial cells	29,416,822
			29,571,080
			31,736,959
Il-36Ra	IL-1F5	Monocytes, KCs, DCs	29,416,822
			29,571,080
			31,736,959

As we known, autoimmune diseases were an ordinary sort of diseases with the malfunction in self-tolerance (Costenbader et al., 2012) It could be classified according to the body system affected. Some cases affected multiple body systems, whereas others affected only one organ. Generally, common autoimmune diseases included psoriasis, (Furue et al., 2018) rheumatoid arthritis (RA), (Wang et al., 2016), inflammatory bowel disease (IBD), (Nishida et al., 2016) systemic lupus erythematosus (SLE), (Mai et al., 2018) neuromyelitis optica spectrum disorder (NMOSD), (Song et al., 2019) primary Sjogren’s Syndrome (pSS), (Ciccia et al., 2015), myasthenia gravis (MG), (Zhang et al., 2020) systemic sclerosis (SSc), (Denton and Khanna, 2017) and so on. Although rapid progress of the treatment in autoimmune diseases has been made in several decades, the exact pathogenesis of autoimmune diseases is still requested to be explored. Pathogeny of autoimmune diseases may include genetic factors, environmental factors, hormones, and so on (Costenbader et al., 2012) On the other hand, inflammation was a common symptom in most autoimmune diseases. Some cytokines especially interleukins have been proved to be used for communication with the immune system (Chen et al., 2006). Additionally, researches demonstrated that IL-36 took part in immune cell activation, antigen presentation, and pro-inflammatory cytokine production (Jati et al., 2019) As the report depicted, peripheric lymphocytes could express IL-36γ through stimulating *α* particle, while T lymphocytes managed to express IL-36 agonists under specified conditions. Therefore, IL-36 could mediate pro-inflammatory activity, whose function was depended on the form and location of inflammation (Ding et al., 2018).

Recently, our research group was committed to studying the relationship between IL-36 and autoimmune diseases. Therefore, this review was to summarize the depth and breadth of current research on this issue. In this review, the pathophysiological roles of IL-36 in different autoimmune diseases will be discussed, especially those related to inflammatory responses. These findings may provide a new direction for the future research of autoimmune diseases.

### Overview of Autoimmune Diseases

A study has shown that there is a link between autoimmune diseases and some degree of biological disorders (Christen, 2019) In another word, autoimmune diseases may be occurred when the immune system cannot play a key role in protecting the host from infectious factors. (Wang et al., 2015) Besides, another argument was that the basis of autoimmune diseases was often termed a breach of tolerance, when the system cannot clearly distinguish between self and non self (Wang et al., 2015; Gershwin, 2018) More and more evidences suggested that the prevalence of autoimmune diseases was increasing (Saccucci et al., 2018) Recently, autoimmune diseases were proved to contain more than 80 chronic diseases (Ortona et al., 2016) and affected up to 10% of the population (Islam et al., 2020) Furthermore, over 50% patients with autoimmune diseases were accompanied by reduced quality of life and related symptoms of depression (Pryce and Fontana, 2017) More specifically, nearly 15 million people in the United States suffered from autoimmune diseases, and this number is increasing every year (Yamamoto and Jorgensen, 2019) Taken together, autoimmune diseases may be one of the risk factors affecting people’s normal life.

The main pathogenesis in autoimmune diseases was the overactivation of autoantibodies, especially when immune system was overrun under the activation of T and B cells (Lee et al., 2020) On the one hand, defects in inflammatory signal resolution increased the risk of autoimmune diseases, and the activation of several inflammatory mediators contributed to the development of autoimmune diseases (Baccala et al., 2007) In particular, when pro- and anti-inflammatory cytokines were imbalanced, tissue damage and inflammatory diseases would be resulted (Abdolmaleki et al., 2020) Moreover, when the immune system failed to resolve inflammation, it would lead to persistence of the inflammatory process in autoimmune diseases (Das, 2011) On the other hand, autoimmune diseases caused various injuries and dysfunctions in multiple organs of the body (Ortona et al., 2016; Lee et al., 2020) For example, some endocrine organs were often attacked targets in autoimmune diseases. Diabetes, thyroiditis, and hypoadrenalism were often caused by immune attack on endocrine cells, which were vital for hormone production (Gershwin, 2018) Previous analysis results provided a strong evidence indicating that 180 gene loci were associated with more than 12 autoimmune diseases. This result directly suggested a relationship between autoimmune diseases and genes. With increasing awareness, gene therapy has become an approach for the treatment of autoimmune diseases (Lee et al., 2020) Recent studies suggested that the interleukin family may be involved in immune regulation. It was well known that IL-10 was an important immunoregulatory cytokine in intestinal mucosal immune homeostasis and mediated immunosuppression by regulatory T cells (Treg cells), which were important mediators of peripheral self-tolerance (Huang et al., 2021) Furthermore, the understanding of the balance between Th1 and Th2 relied on the discovery of IL-27 (Shimizu et al., 2005) Additionally, IL-31 may be a key regulatory cytokine in Th2 responses (Dillon et al., 2004) IL-32 and IL-33 were then key components of the inflammatory response (Dillon et al., 2004; Schmitz et al., 2005) IL-23 produced by macrophages and stem cells in the intestinal epithelium also played a pro-inflammatory role (Almradi et al., 2020).

Moreover, a member of the interleukin family cytokines was highly expressed in *epidermis*, bronchus, gingiva and intestinal epithelium, and may be upregulated in contact with bacterial components. It was suggested that the cytokine had related functions at the barrier interface. Next, a novel cytokine, IL-36, was found to be active in barrier tissues (Queen et al., 2019) Subsequently, the role of IL-36 in modulating the interaction between the environment and the organism was demonstrated, which considered their response to microorganisms, extracellular ligands, and some proinflammatory mediators. Besides, some neutrophils closely related to autoimmune diseases were derived from histone G and elastase, and may affect the activation of IL-36 agonists (Yuan et al., 2019) Interestingly, neutrophil derived extracellular traps (NETs) were later found to be a platform for pro-inflammatory cytokine activation, IL-36 subfamily cytokines could be efficiently processed and activated by net associated cathepsin G and elastase (Clancy et al., 2017) In general, although autoimmune diseases posed a great threat to human physical and mental health, the interleukin family, especially IL-36, may provide a new avenue for further research on autoimmune diseases.

### Overview of IL-36

IL-36 consisted of four members: IL-36α, IL-36β, IL-36γ, and IL-36Ra, which were designed as IL-1F6, IL-1F8, IL-1F9, and IL-1F5. (Table 1) The encoding genes for IL-36 were located on human chromosome 2q13 within a 360 kb region (Ding et al., 2018) In addition, the IL-36Ra could inhibit activation of IL-36R signaling. In another word, IL-36 bound to the IL-36R and used the IL-1 receptor accessory protein (IL-1RAcP) as a co receptor, leading to the activation of intracellular signals (Mai et al., 2018).

**FIGURE 1:**
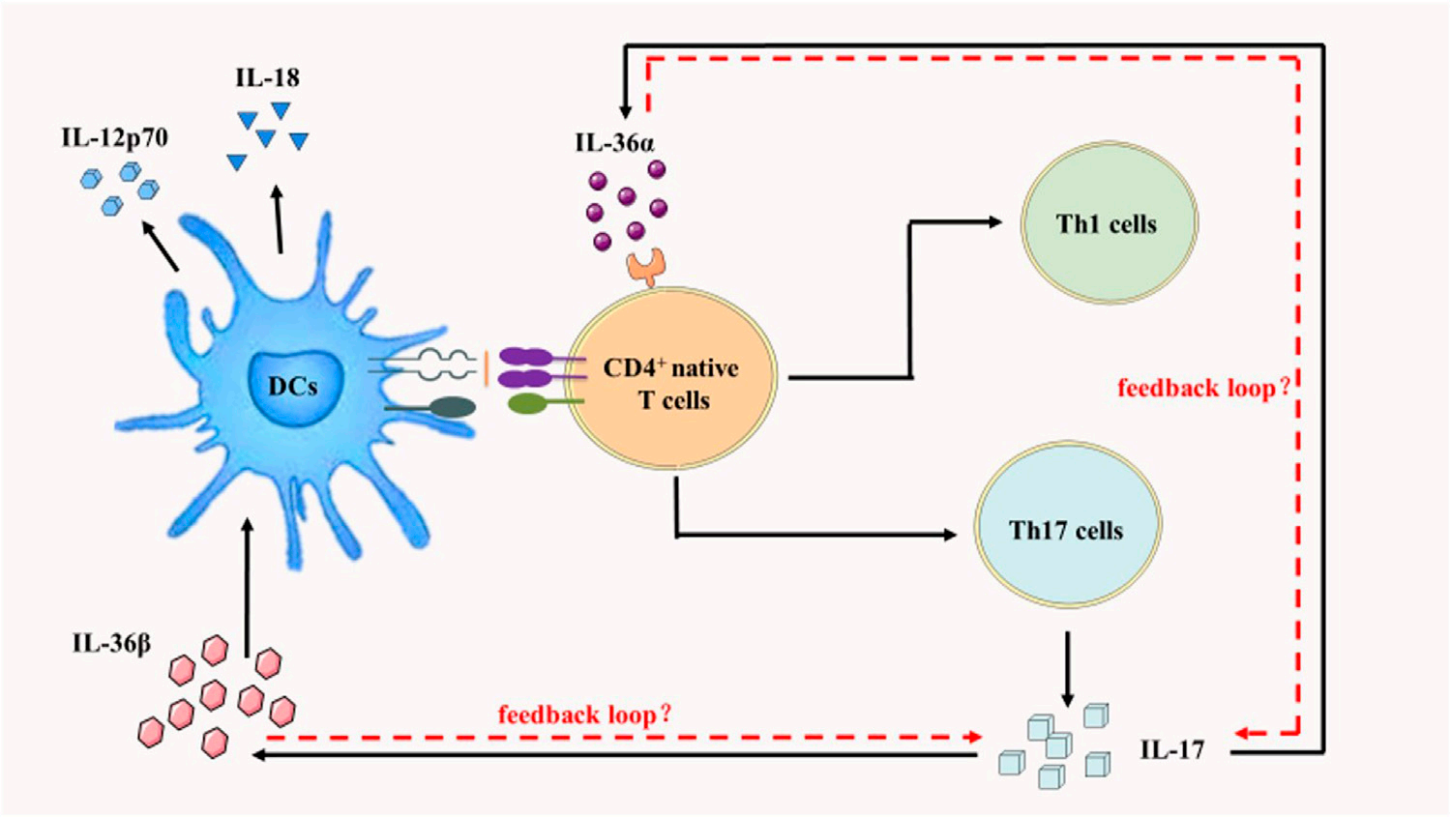
IL-36 and IL-17 may acuate a strong feedback loop which modify skin inflammation. IL-36α stimulated CD4^+^T cells under the polarization of Th1cells. IL-36β upregulated the production of IL-12p70 and IL-18 in MDDCs. IL-17 released by Th17 cells could in return upregulate IL-36 production.

In human KCs, IL-22, IL-17A, and tumor necrosis factor-α (TNF-α) induced the production of IL-36 agonists, whereas interferon-γ (IFN-γ) selectively induced IL-36β production (Yuan et al., 2019) The stimulatory effects of IL-36 were relatively unique, as other IL-1 family cytokines containing IL-1 itself had little effect on mouse bone marrow mesenchymal stem cells (BMSCs) (Gabay and Towne, 2015) Additionally, IL-36 stimulated the production of chemotactic agents that activated leukocytes, and it could promote leukocyte infiltration and cutaneous acanthosis in mice. Excess IL-36β stromal cells were induced to mitotically generate KCs. This led to hyperproliferation of KCs and resulted in hyperplasia of the skin (Queen et al., 2019) Besides, IL-36 has been shown to be an important regulator of mucosal homeostasis and inflammation. Moreover, IL-36 and its proinflammatory cytokines could enhance each other and cause similar responses in KCs (Wang et al., 2017) Strictly speaking, IL-36 couldn’t be considered a proinflammatory cytokine. Because IL-36 upregulated anti-inflammatory ligands while also downregulating the effects of multiple functional components. Thus, IL-36 signal transduction relied on the balance of multiple units in an integrated system. Besides, net activation relied on the cumulative effects of whole components, including extracellular proteases, cytokine ligands, receptor subunits, and downstream cytokines with functional roles. (Swindell et al., 2018) Functionally, IL-36 stimulated the production of several cytokines, chemokines, adhesion molecules, and proinflammatory mediators. (Vigne et al., 2011) Th1 and Th2 expressed relatively high levels of IL-36R mRNA in CD4^+^T cells. Notably, three IL-36 agonists induced production of IFN-γ, IL-4 and IL-17 in CD4^+^T cells and splenocytes. (Mai et al., 2018; Vigne et al., 2011) Furthermore, IL-36 moderately matured DCs. Their upregulated levels of major compatibility complex II (MHCII) antigens and CD40, CD80, and CD86 were comparable to those stimulated by lipopolysaccharide (LPS). In response to IL-36 stimulation, activated DCs could produce IL-1β, IL-6, IL-23, TNF-α, CCL1, CXCL1, and granulocyte colony-stimulating cytokine. These cytokines and chemokines could induce Th1 and Th17 responses. (Mai et al., 2018; Vigne et al., 2011) In other words, IL-36α stimulated CD4^+^T cells under Th1 polarization. This suggested that IL-36α could efficiently drive Th1 responses. IL-36β upregulated the production of IL-12p70 and IL-18 in monocyte derived dendritic cells (MDDCs), suggesting stimulation of the Th1 phenotype. (Yuan et al., 2019) Also, IL-17 released by Th17 cells could in return upregulate IL-36 production. Thus, IL-36 and IL-17 may initiate a strong feedback loop in switching skin inflammation, (Figure 1) (Mahil et al., 2017) suggesting that IL-36 may be primarily involved in activating immune responses. (Queen et al., 2019; Madonna et al., 2019).

According to the international agency for research on cancer, there were approximately 700,000 liver cancer deaths annually worldwide. In China, hepatocellular carcinoma (HCC) was the most common cancer diagnosis. (Sarin et al., 2020) A study results showed that IL-36 was significantly expressed in HCC tumor tissues compared with adjacent peritumoral tissues, and its expression level was closely associated with clinical parameters, such as metastasis, alpha fetoprotein, HBV infection, and cirrhosis, but did not change with age and gender. (Hu et al., 2020) The expression level of IL-36 has also been confirmed to be associated with the prognosis of liver cancer. Specifically, if the expression level of IL-36 was higher, the patients would have a better prognosis of liver cancer and a longer survival time. These suggested that IL-36 may be a biomarker for HCC. (Hu et al., 2020) Besides, although colorectal cancer (CRC) was the third most common cancer in the world, it was relatively common in Western societies (Marley and Nan, 2016), and the incidence has also increased in China (Maida et al., 2017) IL-36 could be produced at high levels in the colon, which was beneficial to the survival of CRC patients. (Wang et al., 2014) In other words, high IL-36α in CRC patients had higher survival than low IL-36α patients. (Chen et al., 2020) However, in colorectal cancer patients, high IL-36β groups and low IL-36β groups were not statistically different, but colonic mucosal IL-36β was reduced by 80% in CRC tissues. This suggested that although IL-36β may not be a good indicator for predicting the prognosis of CRC, but it may be involved in inhibiting the development of this cancer. (Chen et al., 2020).

In addition, IL-36 played a role in the coordination of innate and adaptive immunity, (Saha et al., 2018) specific examples of which would be explained in the following exposition. In recent years, the aberrant expression levels of IL-36 in autoimmune diseases have attracted attention, as well as the correlation between IL-36 and the occurrence and development of diseases. Correspondingly, inhibition of IL-36 holds promise to open new areas in the treatment of autoimmune diseases.

### The Function Roles of IL-36 in Autoimmune Diseases

Damage to tissues and organs in autoimmune diseases mainly manifested as immune response disorders. These responses generated an abnormal antibody host that may damage the body’s own cells and tissues. Despite there were the large number of anticipated treatments for autoimmune diseases, most of the exact causes of autoimmune diseases were unknown. However, recent studies have shown that cytokines were crucial in the pathogenesis of autoimmune diseases, particularly the dysregulation of IL-36 in various autoimmune diseases (Table 2).

**TABLE 2:** The serum level and the gene level of segmental IL-36 ligands.

Autoimmune diseases	Serum level of segmental IL-36 ligands	Expression level of segmental IL-36 ligands	References
Psoriasis	Increase	Increase	29,757,591
			28,967,976
RA	Increase	—	27,188,731
IBD	—	Increase (except IL-36β)	26,752,465
SLE	Increase and high	Low	29,571,080
NMOSD	Increase	—	3,0,852,049
			31,382,910
pSS	Increase	Increase (except IL-36R)	25,902,739

### IL-36 and Psoriasis

Psoriasis, a papulosquamous skin disease, was considered one of the most common immune-mediated diseases. (Griffiths and Barker, 2007) Psoriasis presented different prevalence in different populations, (Furue et al., 2018) which affected about 3% of 30-year-old American adults and 0.1% of children and adolescents. (Wang et al., 2017; Korman, 2020) Immune system disorder was an important factor in the pathological mechanism of psoriasis. This was because psoriatic lesions were the result of interactions between dysregulation of resident skin cell types and innate and adaptive components of the immune system. (Boehncke and Schon, 2015) Previous studies have shown that TNF-α/IL-23/IL-17/1L-22 axis played a crucial role in the development of psoriasis. (Furue et al., 2018) Recently, the potential attenuation of skin inflammation in an IL-23-induced psoriasis mouse model was investigated by functional inhibition of IL-36R, which confirmed that functional activation of IL-36R was also one of the driving forces of psoriasis (Su et al., 2019).

Mojtaba Sehat et al. found that serum IL-36 level was increased in psoriasis. (Sehat et al., 2018) The gene expression level of IL-36 was also increased in psoriasis. (Furue et al., 2018) Besides, the removal of one or two copies of the IL-36Ra gene would aggravate the severity of the phenotype. Furthermore, mice with forced overexpression of IL-36a in the skin was created, and a similar skin inflammatory condition existed in human psoriasis. Skin disorders included thickened squamous skin, acanthosis, hyperkeratosis, and inflammatory infiltrates in the dermis. (Towne and Sims, 2012) Macrophages in psoriasis driven by activating IL-36γ and IL-36R produced IL-23 and TNF-α. This effect was specific for IL-36γ and cannot be mimicked by other IL-1 family cytokines. These diverse experimental results basically suggested a potentially powerful biomarker IL-36 in psoriasis. Moreover, IL-36 affected the function of macrophages, which contributed to the pathogenesis of psoriasis. High levels of IL-36R and IL-36 specifically affected anti-inflammatory M2 macrophages, rendering them a pro-inflammatory M1 phenotype. (Madonna et al., 2019) Therefore, to further elucidate the biological importance of IL-36 in psoriasis, a study performed IL-36α, IL-36β, and IL-36γ gene enrichment analysis of the transcriptome. (Mahil et al., 2017) Although the most indispensable functional role was that of IL-17A in psoriasis, 26 (56%) of the 46 genes upregulated by IL-17 in KCs belonged to the IL-36 group. This further indirectly confirmed that IL-36 may play an important role in psoriasis. (Mahil et al., 2017) It was worth noting that another mechanism to enhance Th17 activity was also related to IL-36, implying induction of phospholipase A2 group IVD (PLA2G4D). (Mahil et al., 2017) The findings demonstrated the enhanced expression of PLA2G4D in psoriatic *epidermis*, which was comparable to normal skin and eczematous lesions. (Ten Bergen et al., 2020) Besides, mast cells, as one of the immune cells, were the main cell source of PLA2G4D, so it was not difficult to speculate the relationship between PLA2G4D and immune diseases. IL-36 could upregulate phospholipase PLA2G4D, which generated lipid antigen-presenting CD1A reactive T lymphocytes. Stimulation of this cell led to the production of IL-17 and IL-22 (Ten Bergen et al., 2020).

Taken together, reducing IL-36 may be another option for anti-IL-17α therapy in psoriasis. Overall, there may be other regulatory mechanisms between IL-36 and various cytokines in psoriasis.

### IL-36 and RA

RA was a systemic inflammatory diseases that primarily affected the diarthrodial joint. (Lee and Weinblatt, 2001) It infected 0.5–1.0% of adults in developed countries, and the disease was typically three times more common in women than in men. RA was defined as a clinical syndrome bridging disease subsets. These different subsets required some inflammatory cascades that ultimately led to a common pattern. The modality was persistent synovial inflammation and was associated with articular cartilage and underlying bone disease. (Scott et al., 2010) Inflammatory cytokines also seemed to be included in the pathological mechanism of RA. (Wang et al., 2016).

Recent studies confirmed the serum IL-36α and IL-36β levels were upregulated in RA patients, and the upregulation between IL-36α and C-reactive protein (CRP) were positively correlated. (Wang et al., 2016) It was reported that subgroups of RA could potentially raise IL-36 agonist/antagonist ratio. (Boutet et al., 2016) Although IL-36α was expressed in a variety of cells, plasma cells were the major source of IL-36 in arthritis. This was because that IL-36α may play a key role in autoimmune and inflammatory responses, which was manifested by plasma cell infiltration into the synovium. (Mai et al., 2018) Besides, IL-36α was a major pro-inflammatory trigger. IL-6 (which stimulated the composition of TNF-α) and IL-8 (a major chemoattractant molecule in which leukocytes migrated further into joints) were markedly upregulated in fibroblast like synoviocytes (FLS). Through this novel mechanism, plasma cells could interact with FLS residents in inflammatory arthritis. In addition, upregulation of IL-36 was related to the presence of inflammatory infiltration in synovial tissue. (Frey et al., 2013) Furthermore, IL-36 was associated with IL-1β, IL-1β, CCL3, CCL4, and macrophage colony stimulating factor (MCSF) in collagen-induced arthritis mice and RA patient synovium, but not with Th17 cytokines. (Boutet et al., 2016) Notably, reduction of IL-36 cytokines didn’t alter the histological signs of TNF-induced arthritis. Similarly, scientists didn’t find differences in the levels of pro-inflammatory cytokines in mice by decreasing IL-36 (Derer et al., 2014).

Although IL-36 has a pathological effect in RA, it is crucial to understand more about the role of IL-36 in other diseases. Therefore, it is possible to confirm its immunological potential in the disease.

### IL-36 and IBD

IBD was a chronic intestinal inflammation with a relapsing course. It included ulcerative colitis (UC) and Crohn’s disease (CD). (Friedrich et al., 2014) More than a decade ago, results from a population-based pediatric study of IBD showed an annual incidence of 4.56 per 100,000 children for CD and 2.14 per 100,000 children for UC. Recent findings indicated that these rates appeared to be rising. (Shapiro et al., 2016) Besides, IBD was characterized by an imbalance between innate and adaptive immunity. This imbalance may stimulate T helper responses with a preponderance of pro-inflammatory cytokines. (Friedrich et al., 2014) Studies pointed out that new cytokines, such as IL-36, might be considered useful tool to predict disease progression.

The mRNA expression level of IL-36α and IL-36γ, but not of IL-36β, was increased in the inflamed mucosa of IBD patients, particularly in UC. (Nishida et al., 2016) In dextran sulfate sodium induced colitis and in the colon of CD patients, IL-36α and IL-36γ levels were correspondingly lower and comparable to IL-1β and IL-17A. (Boutet et al., 2016) Differentiation proteins of IL-36α, IL-36β, IL-36γ, and IL-36Ra, intestinal epithelial cells, macrophages, CD8^+^T cells, and/or DCs were also overexpressed in patients with active IBD. (Fonseca-Camarillo et al., 2018) This suggested that IL-36 could be considered as another potential tool for the diagnosis of IBD activity, and the differences in IL-36β activity could be used to distinguish UC from active IBD. Interestingly, although IL-36α and IL-36γ lacking canonical protease cleavage sites, many extracellular neutrophils could effectively modulate the activity of these cytokines through processing. Thus, activated neutrophils released multiple proteases into extracellular space. If these proteases presented locally, they could be enzymatically converted and could activate the IL-36R ligand. (Neufert et al., 2020) What’s more, IL-36α and IL-36γ might play a pro-inflammatory role in pathological mechanisms of IBD by stimulating CXC chemokines (CXCL1, CXCL2, CXCL3, and so on) and acute phase proteins. (Nishida et al., 2016) IL-36Ra was showed to reduce inflammation by blocking binding of receptor ligands. Thus, inhibition of IL-36R could produce anti-inflammatory effects. It could also induce antifibrotic signals. (Fonseca-Camarillo et al., 2018) Moreover, the differentiation of T cell was influenced by IL-36R activation driving pro-inflammatory Th1 and Th9 responses. Correspondingly, IL-36R axis controlled the balance between the anti-inflammations and pro-inflammatory Th9 cells of regulatory T cell (Neufert et al., 2020).

However, the functional roles associated with IL-36Ra remains unclear, and further studies are needed to explain its role in IBD. Therefore, innovative and rational targets can be provided for therapy.

### IL-36 and Systemic Lupus Erythematosus

SLE was a chronic autoimmune disease, which had a range of clinical differences and abnormalities of the immune system by producing autoantibodies. This response targeted nuclear and cytoplasmic antigens and then affected several different organs. (Fortuna and Brennan, 2013) SLE had the highest estimated incidence in North America at 23.2 cases per 100,000, whereas it was more common in African Americans, Hispanics, and Asians than in whites (Kiriakidou and Ching, 2020) Besides, SLE was characterized by dysregulation of activation in both T and B cells, followed by overproduction of autoantibodies and proinflammatory cytokines. Dysregulated cytokines were prevalent in SLE, such as imbalanced anti-/pro-inflammatory cytokine profiles and skewed agonist/antagonist levels. This might be part of a major process in lupus pathogenesis (Chu et al., 2015).

Serum IL-36Ra level was downregulated in SLE patients, whereas serum levels of IL-36α and IL-36γ were elevated and correlated with complement C3 levels. (Mai et al., 2018) Studies further found that serum levels of IL-36α and IL-36γ were positively correlated with increased levels of IL-10, which was considered as a biomarker of SLE. This suggested that IL-36 played a role in progression of SLE, particularly in end-stage diseases. (Chu et al., 2015) Interestingly, IL-36α levels were significantly higher in SLE patients, but lower mRNA levels. (Mai et al., 2018) There are several possibilities: 1) the higher translation efficiency of IL-36α; 2) the reduced rate of protein degradation; 3) the rapid secretion of IL-36α; 4) the negative feedback from controls in gene or protein, or may indicate other noncomplementary regulators. (Mai et al., 2018) Therefore, this mechanism should be further explored.

### IL-36 and Neuromyelitis Optica Spectrum Disorder

NMOSD was an inflammatory central nervous system (CNS) syndrome typically associated with serum aquaporin-4 immunoglobulin G antibodies (AQP4—IgG). (Wu et al., 2019) The incidence and prevalence of NMOSD were depended on geographical location and ethnicity, with a higher risk in Asians and Africans. More importantly, approximately 50% of untreated NMOSD patients would become wheelchair users and blind. One-third of NMOSD patients, if untreated, would die within 5 years of their first episode. (Huda et al., 2019) Although anti-aquaporin-4 antibodies (AQP4-ABS) were strongly implicated in the pathogenesis of NMOSD, some studies also suggested that IL-36 may play some role in NMOSD.

In recent studies, serum IL-36 agonists levels were enhanced in NMOSD patients. This study proved that IL-36γ from neutrophils could stimulate microglia to produce neutrophil-stimulating cytokines, and may contribute to neuroinflammation by promoting neutrophil-recruitment (Song et al., 2019; Yang et al., 2019) Neutrophils played an important role in the pathological mechanisms of NMOSD, because polymerization of abnormal neutrophils was found in NMOSD lesions, and the inhibition of neutrophilic protease could attenuate AQP4-IgG damage in the mouse brain (Yang et al., 2019) In addition, murine bone-marrow derived dendritic cells (BMDC) and CD4^+^T lymphocytes both expressed IL-36R and responded to IL-36α (Song et al., 2019) In turn, the increase of IL-36α may affect immune cells in NMOSD and cause ongoing inflammation. There was a similar cytokine milieu in the cerebrospinal fluid of NMOSD patients and in the synovial fluid of patients with RA. Furthermore, IL-36α may locally ameliorate the effects of IL-17α and TNF-α, and contributed to the pathomechanisms of psoriasis (Yang et al., 2019).

Though the role of IL-36 in neuromyelitis remains to be studied, IL-36 may be involved in the pathologic mechanisms of NMOSD. Also, IL-36α-mediated inflammation may be associated with NMOSD, and therefore IL-36α may be a new biomarker for monitoring the severity of NMOSD.

### IL-36 and primary Sjogren’s Syndrome

PSS was an autoimmune disease that mainly affected exocrine glands, such as the salivary and lacrimal glands. (Bjordal et al., 2020) It may affect 2 per 1,000 patients per year, and the overall prevalence in Europe was close to 1%. (Ramos-Casals et al., 2012) Multiple activations of the innate immune system and B and T lymphocytes in pSS were related to disease, whereas IL-36 seemed to be the essential control axis (Boutet et al., 2016).

Serum IL-36α level was higher in pSS and IL-36α expression was significantly increased in pSS salivary gland tissue. (Ciccia et al., 2015) However, IL-36R expression levels were not increased. Studies demonstrated that both IL-36Ra and IL-38 were inhibitors of IL-36α in pSS, but IL-36Ra downregulated and IL-38 overexpressed. (Ciccia et al., 2015) It suggested that the immune system attempted to neutralize the imbalance in IL-36 activation. (Ciccia et al., 2015) What’s more, IL-36 had been implicated in the pathogenesis and the regulation of γδ^+^ immune function of T cells. (Ciccia et al., 2015) In pSS patients, a higher proportion of γδ^+^ T cells co-expressed IL-17, IL-36α, and IL-36R. (Ciccia et al., 2015) It may indicate a possible autocrine stimulus circuit. Therefore, the mechanism of IL-36 on pSS should be further studied.

### IL-36 and Myasthenia Gravis

MG was a disorder of neuromuscular transmission leading to fatigue and fluctuating weakness of skeletal muscles. (Peragallo, 2017) MG could present at any age with a prevalence of 100–200 per 1 million people per year. But there were usually two peaks: in the second to early thirties (female-dominated) and in the late eighties (male-dominated). (Martinez Torre et al., 2018) MG was an antibody-mediated inflammatory disease. Antibodies mainly destroyed acetylcholine receptor (AChR) at the neuromuscular junction. Recent studies confirmed a link between IL-36 and MG. It demonstrated that serum level of IL-36γ was up-regulated in MG patients. Interestingly serum IL-36γ level in patients with ocular myasthenia gravis (OMG) was lower than those with systemic myasthenia gravis (GMG). (Zhang et al., 2020) However, the serum levels of IL-36α and IL-36β had no difference. (Zhang et al., 2020) This may indicate that IL-36γ was positively correlated with the severity of MG. Thus, the distinct pathogenesis of IL-36 in MG should be further investigated.

### IL-36 and Systemic Sclerosis

SSc, also known as scleroderma, was an immune-mediated rheumatic disease. (Denton and Khanna, 2017) It was characterized by dermal and visceral fibrosis and vascular lesions. It was estimated to affect 1 in 10,000 people worldwide. Importantly, mortality in SSc was higher than any other rheumatic disease. Although SSc was generally considered as a typical fibrotic disease within autoimmune rheumatic family, this concept may be an oversimplification. The pathogenesis of SSc was complex, and incompletely understood. But the studies of IL-36α showed that this variant could promote inflammation and fibrosis. (Xu et al., 2019) Firstly, IL-36α was found to promote tubulointerstitial fibrosis in mice with unilateral ureteral obstruction (UUO). Secondly, elevated IL-36α and IL-1R6/IL-1R3 receptor complex was demonstrated in myofibroblasts. It was associated with fibrotic tissue in patients with chronic pancreatitis. (Artlett, 2018) Finally, NLRP3 gene transcription was increased in SSc fibroblasts. Moreover, in skin biopsy samples from SSc patients, it proved that NLRP3 inflammasome hyperactivation and positively correlated with skin thickness. More importantly, recombinant IL-36α could stimulate the NLRP3 inflammasome. (Xu et al., 2019) In conclusion, more studies are suggested to clarify the role of IL-36 and its receptor in fibrosis.

### Regulatory Role of IL-36 in Autoimmune Diseases

**FIGURE 2:**
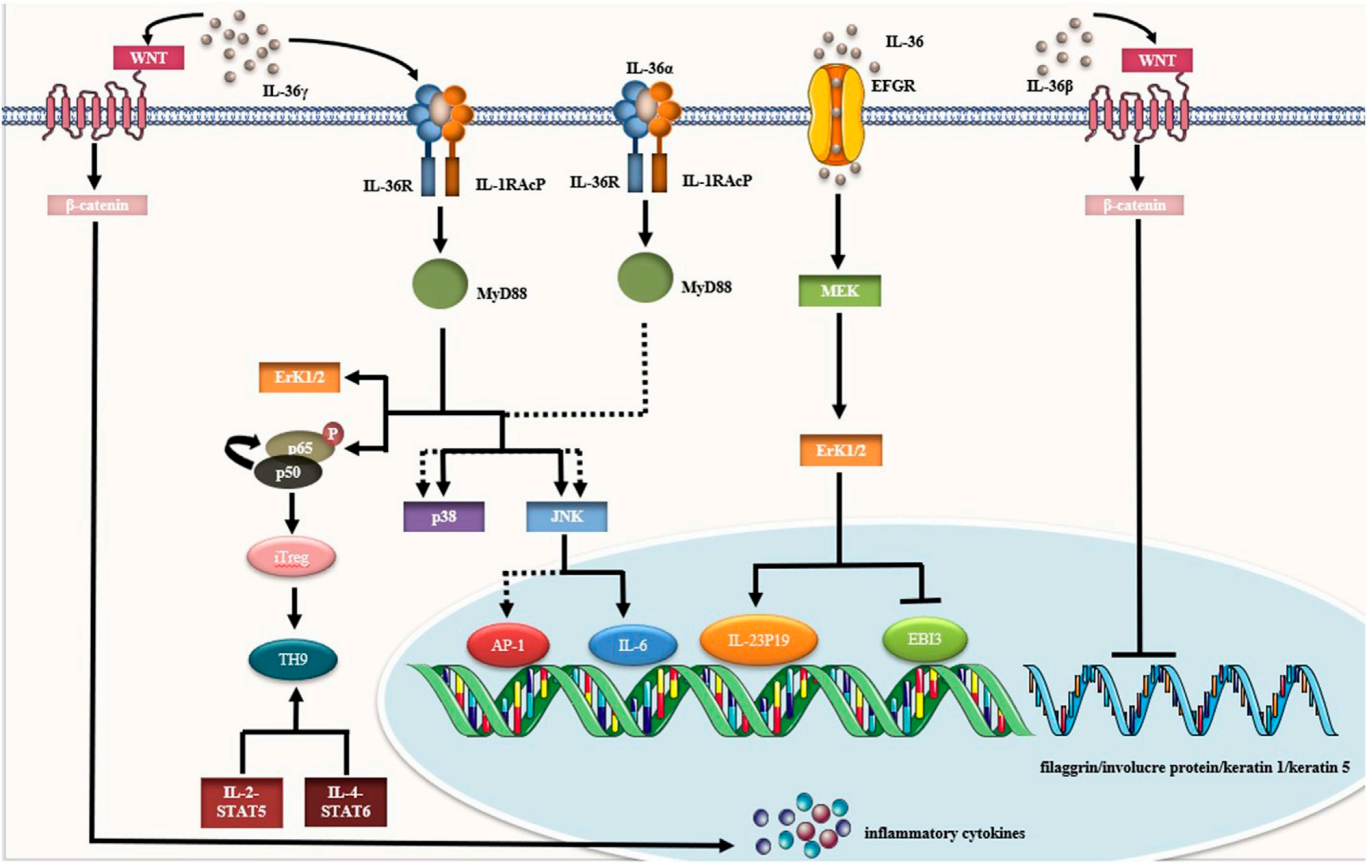
IL-36 ligands induced different signaling pathways, which may promote or inhibit pro-inflammatory response. Common signaling pathways included JNK, MEK-ERK, MAPK-p38, Wnt signaling pathway and so on.

It was well known that complex molecular mechanisms were included in the occurrence and severity of autoimmunity. Current understanding was incomplete, so it was difficult to treat them effectively. However, it has been recognized that IL-36 may be a potent biomarker for autoimmune diseases through direct or indirect regulation. Thus, this section summarized the cellular mechanisms of IL-36 in autoimmune diseases. (Figure 2).

### Dectin-1/SYK Signaling Pathway

The Dectin-1/SYK signaling pathway could interact with the TLRS-dependent MyD88 signaling pathway to enhance the cytokine release. (Dennehy et al., 2008) Additionally, Dectin-1/SYK and TLR4 signaling pathways have been shown to be involved in the induction of IL-36γ. (Gresnigt et al., 2013) The interaction between Dectin-1 receptor and TLR2 enhanced the MAPK signaling pathway. It could enhance cell phagocytosis of fungi and drive Th17 response. It also increased the cytokine production. (Goodridge and Underhill, 2008) TLR2 and CR3 ameliorated negative regulation of IL-36γ induced by Aspergillus. Previous experiments couldn’t detect the generation of IL-17 or IFN-γ when IL-36γ or IL-36β was bound to IL-12 or IL-23. This was contrary to the binding of IL-1/IL-23 and IL-18/IL-12, suggesting that IL-36 ligand was not a substitute for IL-1β or IL-18 (Gresnigt et al., 2013).

### MEK-ERK Signaling Pathway

The MEK-ERK signaling pathway could regulate IL-36γ-induced expression of IL-23p19 and EBI3 through host factors (such as growth factors). Epidermal growth factor (EGF) activated MEK-ERK signaling pathway in epithelial cells, enhanced the activation of IL-23p19 expression, and inhibited EBI3 expression. IL-23p19 and EBI3 were a heterodimerization to form a new cytokine-IL-39 that could improve neutrophil elongation (Scholz et al., 2018).

### Wnt Signaling Pathway

The Wnt signaling pathway was mainly involved in embryonic development and self-renewal of adult tissues. It could exert anti-inflammatory and pro-inflammatory effects through multiple mechanisms. β-catenin was a major regulator in the Wnt signaling pathway. In KCs, IL-36γ induced release of inflammatory cytokines (such as IL-1β, IL-6, and IFN-γ) through the Wnt signaling pathway. Also, IL-36β enhanced down-regulation of filaggrin, involucre protein, keratin 1, and keratin 5 at mRNA levels by inhibiting differentiation of KCs (Wang et al., 2017).

### Other Signaling Pathways

Firstly, stimulation of IL-36R promoted the recruitment of MyD88-binding proteins to the TIR domain. (Swindell et al., 2018). IL-36α could rapidly induce the composition of MyD88 linked molecules to form complexes and activated JNK, MAPK, and ERK1/2 signaling pathways. This complex consisted of MyD88, TRAF6, IRAK1, and TAK1. IL-36R could also induce phosphorylation of MAPKs, and then activated p42/44MAPK, p38MAPK, and JNK signaling pathways. (Nishida et al., 2017; Swindell et al., 2018; Li et al., 2019) IL-36γ activated the JNK signaling pathway through IL-36R/IL-1RAcP. This might lead to the secretion of IL-6. In other words, blocking the JNK signaling pathway could inhibit upregulation of IL-6 and IL-2 induced by IL-36γ *in vitro* (Meissner et al., 1988) In fact, the acute response phase was activated by IL-6, which was identified as the transcriptional regulator STAT3. STAT3 was then regulated by IL-17, IL-22, IL-23, and other cytokines. (Lichawska-Cieslar et al., 2021) Besides, the differentiation of Th9 cell was simultaneously promoted through IL-2-STAT5-dependent and IL-4-STAT6-dependent signaling pathways. (Harusato et al., 2017) Previous studies have shown that rapid induction of the MyD88 complex by IL-36α caused the activation of NF-κB/(Activator Protein 1) AP-1. (Swindell et al., 2018) MAPK signaling pathway inhibitors inhibited the activation of c-Jun (AP-1), but not NF-κBp65. It further confirmed that NF-κB was activated in a similar manner by IL-36α stimulation, whereas AP-1 was activated by IL-36α in a tandem manner. Furthermore, NF-κBp65 and c-Jun-specific siRNAs transfection inhibited IL-36-induced secretion of CXCL1, CXCL8, matrix metalloproteinase-1 (MMP-1) and matrix metalloproteinase-3 (MMP-3). (Nishida et al., 2017) IL-36γ promoted nuclear transfer of NF-κBp65 and p50 under neutral conditions, but not p105. (Harusato et al., 2017) Enhanced nuclear translocation of NF-κBp65 may alter the overall proportion of NF-κBp50 to NF-κBp65 via IL-36. It led to impaired differentiation of iT_reg_ cells. Moreover, IL-36R inhibited the differentiation of iT_reg_ cells through MyD88 and NF-κBp50 signaling pathways in CD4^+^T cells, and redirected to IL-9-producing iT_reg_ cells. Additionally, IL-36γ induced expression of mucin 5Ac in human airway epithelial cells through IL-36R-mediated p38-NF-κB signaling pathway (Harusato et al., 2017).

### The Therapeutic Potential Role of IL-36 in Autoimmune Diseases

Over the years, many studies demonstrated the diagnostic value of IL-36. Taking generalized pustular psoriasis (GPP) as an example, *IL36RN* mutations were common in GPP patients, especially in patients without associated plaque psoriasis. The proportion of *IL36RN* mutation in GPP ranged from 23 to 37%. Disease severity was influenced by *IL-36RN* mutation status. In the analysis of GPP patients, the presence of recessive *IL36RN* alleles (found in 21% of cases), early onset (in patients with no pathogenic *IL36RN* alleles, *p* = 5.9 × 10–7), systemic inflammation (83 vs. 56%, *p* = 1.5 × 10–3), no complications of plaque psoriasis (36 vs. 69%, *p* = 5.0 × 10–4). (Furue et al., 2018) Using IBD as another example, IL-36β-producing potential lymphocytes in submucosal, muscle, serosal, and perivascular tissue from patients with active CD, compared with non-inflammatory control tissue, increased inflammatory infiltration (Fonseca-Camarillo et al., 2018) Moreover, the gene expression of IL-36α (*p* = 0.050), IL-36β (*p* = 0.032 and *p* = 0.036), IL-36γ (*p* = 0.02 and *p* = 0.03), and IL-36Ra (*p* = 0.006 and *p* = 0.007) was significantly higher in colonic inflammatory tissue with active UC compared with inactive UC and non-inflamed controls (Fonseca-Camarillo et al., 2018) Together, these findings statistically demonstrate the potential of IL-36 as a diagnostic biomarker.

Currently, the treatment of autoimmune diseases mainly relied on non-specific immunosuppression (Wu et al., 2020) Despite there were various important breakthroughs in therapeutic medicine, especially with respect to biologics, insufficient selection of targets often led to suboptimal effects, systemic adverse effect and patient nonadherence. The mechanisms of other autoimmune diseases were still being explored. However, by gaining multiple mechanistic and therapeutic knowledge of IL-36 in autoimmunity, it paved the clinical way for inhibitory molecules. Due to its important regulatory role in the inflammatory cascade and humoral innate immunity, IL-36 held promise as a diagnostic biomarker for autoimmune diseases. For example, in a mouse model of psoriatic inflammation induced by imiquimod (TLR7 agonist), the epidermal and skin features of psoriasis were strongly suppressed in IL-36R deficient mice and enhanced in *IL36RN* deficient mice, (Furue et al., 2018) suggesting that reducing IL-36 cytokines also had a potential role in therapy. Similarly, Qian Li’s team established a complete Freund’s adjuvant (CFA) mouse model, and the level of IL-36γ protein in the spinal cord was upregulated from day 1 to day 7 after CFA injection. This phenomenon was consistent with the changing trend of IL-36γ mRNA (*p* < 0.05). IL-36R protein levels were upregulated on day 3 after CFA injection and remained high until final examination on day 7 (*p* < 0.05). To discover the role of IL-36 in CFA-induced inflammatory pain, Qian Li et al. injected IL-36Ra intrathecally on the seventh day after the injection in CFA. The results confirmed that hyperalgesia improved from day 2 of IL-36Ra treatment and persisted until the last IL-36Ra treatment (*p* < 0.05). Furthermore, IL-36Ra treatment for 5 days reduced mechanical pain abnormalities continuously with 65% recovery in mice after the last injection of IL-36Ra (*p* < 0.05). (Li et al., 2019) In conclusion, the accumulation signs provide a rationale for the interference of IL-36 as a potential target for the treatment of autoimmune diseases.

Additionally, serum IL-36 levels were positively correlated with disease severity in psoriasis (Sehat et al., 2018) Besides, serum levels of IL-36α (Song et al., 2019) and IL-36β (Yang et al., 2019) in NMOSD patients correlated with disease severity. Thus, in the future, IL-36 may serve as a prognostic marker in partial autoimmune diseases. Certainly, these still need to continue exploration.

### Future Expectations

IL-36 plays an important role in the initiation and progression of autoimmune diseases. With more in-depth studies of the mechanism of IL-36 and the use of the latest technology to study the relationship between IL-36 and diseases, the understanding of regulatory network of the gene expression will be improved to a new level.

Firstly, immune cells have been a major focus of genome exploration. Remarkably, CRISPR is a major technology used by immunologists to manipulate immune cell genomes to reveal the genetic basis of immunity. (Simeonov and Marson, 2019) CRISPR can be a flexible or a modular system. The former is represented by its use in cell lines, primary human cells, and animal models to knock out gene function or to insert new genetic sequences. The latter is that CRISPR recruits diverse effector functions to specific sites in the genome in a programmed manner. Recently, there is a focus on improving the editing efficiency of CRISPRCas9 in primary human immune cells, rewriting the single gene diseased variant to cure patients with immune related diseases. (Chang et al., 2015) For example, sibling families caused by recessive mutation in IL-2Ra with different autoimmune manifestations could have Foxp3^+^ T_reg_-like cells. (Roth et al., 2018) These cells with dysfunction expressed inappropriate level of IL-2Ra. Non-viral CRISPR based genomic targeting has been shown to correct pathogenic mutations of IL-2Ra and to preserve IL-2Ra expression in T cells from these patients. Although IL-36 belonged to IL-1 family, which was different from IL-2 family, IL-36, IL-2, and IL-4 (Nguyen et al., 2020) in IL-2 family could also be regulated by STAT signaling pathways. (Harusato et al., 2017) Therefore, if gene editing is combined with IL-36, it may have the same effect.

Secondly, anti-cytokine therapy has emerged as a treatment for some autoimmune diseases. In particular, the success of anti-cytokine therapies in the treatment of chronic plaque psoriasis holds the promise for generalizing this approach to other cytokine targets for new drug development. (Veilleux and Shear, 2017) For example, CNTO-1275, the anti-p40 monoclonal antibody against IL-12 and IL-23 was in phase II clinical trials for psoriasis. As in the previous phase I, psoriasis area and severity index decreased by 75% or better in all patients at the highest dose level. There were many studies on the application of this therapy in other immune diseases. For instance, in the open trial of anakinra (IL-1Ra) for SLE, anakinra treatment showed safety and improved arthritis in all four patients. (Anolik and Aringer, 2005) Ustekinumab was a monoclonal antibody targeting the shared P40 subunit common to IL-12/23. (Jefremow and Neurath, 2020) It was approved for the treatment of moderate and severe CD and UC. (Almradi et al., 2020) In a previous introduction, the summary of IL-36 was suggested as a potential drug target for psoriasis and other autoimmune diseases. The functional associations between IL-36 and several cytokines were also summarized. A recent phenotypic study identified a large number of individuals without a functional IL-36R.These individuals retained normal immune function, which may indicate the possibility of treatment by blocking IL-36. (Bridgewood et al., 2018) Thus, it is reasonable to suspect the translational potential of IL-36 for drug evolution in psoriasis and a wide range of autoimmune and/or autoinflammatory conditions.

Finally, IBD, including CD, and UC, is an abnormal mucosal immune response to the gut microbiota. (Larabi et al., 2020) Fortunately, models of congenital colitis further demonstrate the complex relationship between gut microbiota and intestinal inflammation. Studies have found that genetic, environmental, and immune-mediated microbiota interacted in the pathomechanisms of IBD. Genetic mutations and environmental factors are predisposing factors that lead to impaired immune responses to the gut microbiota. This may lead to a pro-inflammatory state. (Glassner et al., 2020) A recent study has shown that the amelioration of inflammation was associated with IL-6, which may be regulated by the intestinal microbiota. (He et al., 2019) Other studies confirmed the mutually beneficial relationship between microbiota and immune cells in *Il10* ^*−/−*^mice. *Il10* ^*−/−*^ mice did not develop typical spontaneous enterocolitis when they were born and maintained under green fluorescence conditions. Similar effects were observed when *Il10* ^*−/−*^ mice were treated with antibiotics from the neonatal period. (He et al., 2019) Other interleukin families may also influence the onset and progression of IBD by affecting intestinal flora. On the one hand, intestinal flora colonization in mice by IBD donors may aggravate colitis immune responses by changing intestinal flora. (Glassner et al., 2020) On the other hand, the important role of IL-36 in IBD was summarized before. (Fonseca-Camarillo et al., 2018) Therefore, there may also be some inflammatory feedback mechanisms between IL-36 and intestinal microbiota in IBD.

## Conclusion

Aberrant expression levels of IL-36 were found in many autoimmune diseases. Although pharmacotherapy targeting IL-36 has made some progress in a few areas such as psoriasis. Most studies of autoimmune diseases were still limited to animal experiments. Th17 cells (IL-17A and IL-22) and Th1 cells (IFN-γ and TNF-α) were proved to play a role in the pathological mechanisms of inflammatory diseases. IL-36 was focused on inducing the generation of Th1 and Th17 cells. In return, they potentiated the effects of IL-36 in an autocrine manner. In addition to being involved in the regulation of skin inflammation, IL-36 was also involved in the inflammatory state of lung tissue, the synovium of joints, and mucosal tissue of the colon. It might first ameliorate the inflammatory response in skin lesion, arthritis, and bowel diseases. But the functional and regulated roles of IL-36 remain unclear. Especially in human, the exact roles of IL-36 in autoimmune diseases requires further investigations.
